# Migrating from partial least squares discriminant analysis to artificial neural networks: a comparison of functionally equivalent visualisation and feature contribution tools using jupyter notebooks

**DOI:** 10.1007/s11306-020-1640-0

**Published:** 2020-01-21

**Authors:** Kevin M. Mendez, David I. Broadhurst, Stacey N. Reinke

**Affiliations:** Centre for Integrative Metabolomics & Computational Biology, School of Science, Edith Cowan University, Joondalup, 6027 Australia

**Keywords:** Metabolomics, Partial least squares, Artificial neural networks, Machine learning, Jupyter, Variable importance in projection

## Abstract

**Introduction:**

Metabolomics data is commonly modelled multivariately using partial least squares discriminant analysis (PLS-DA). Its success is primarily due to ease of interpretation, through projection to latent structures, and transparent assessment of feature importance using regression coefficients and Variable Importance in Projection scores. In recent years several non-linear machine learning (ML) methods have grown in popularity but with limited uptake essentially due to convoluted optimisation and interpretation. Artificial neural networks (ANNs) are a non-linear projection-based ML method that share a structural equivalence with PLS, and as such should be amenable to equivalent optimisation and interpretation methods.

**Objectives:**

We hypothesise that standardised optimisation, visualisation, evaluation and statistical inference techniques commonly used by metabolomics researchers for PLS-DA can be migrated to a non-linear, single hidden layer, ANN.

**Methods:**

We compared a standardised optimisation, visualisation, evaluation and statistical inference techniques workflow for PLS with the proposed ANN workflow. Both workflows were implemented in the Python programming language. All code and results have been made publicly available as Jupyter notebooks on GitHub.

**Results:**

The migration of the PLS workflow to a non-linear, single hidden layer, ANN was successful. There was a similarity in significant metabolites determined using PLS model coefficients and ANN Connection Weight Approach.

**Conclusion:**

We have shown that it is possible to migrate the standardised PLS-DA workflow to simple non-linear ANNs. This result opens the door for more widespread use and to the investigation of transparent interpretation of more complex ANN architectures.

**Electronic supplementary material:**

The online version of this article (10.1007/s11306-020-1640-0) contains supplementary material, which is available to authorized users.

## Introduction

Within a biological system, metabolite concentrations are highly interdependent (Dunn et al. 2011). As such, the usefulness of multivariate data analysis in metabolomics stems from the need to extract biological information from inherently complex covariant data, where metabolite interaction is as important as individual changes in concentration. Historically, partial least squares (PLS), a.k.a. projection to latent structures (Wold 1975; Wold et al. 1993), has been the standard multivariate machine learning (ML) method used to construct predictive models to classify metabolite profiles. The underlying theory of PLS, and its utility to metabolomics, has been documented many times (Geladi and Kowalski 1986; Gromski et al. 2015; Wold et al. 1993, 2001). A key benefit of PLS is the ability to visualise (via a latent variable score plot) the projected metabolomic relationship (clustering) between individual samples before classification.

There are many machine learning (ML) alternatives to PLS, several of which have been applied to metabolomics data. The most popular include support vector machines (Steinwart and Christmann, 2008), random forests (Breiman 2001), and artificial neural networks (Bishop 1995; Wilkins et al. 1994); however, despite coexisting for a similar length of time, none of these methods have gained the popularity of PLS. A survey of publications listed on the Web of Science using the keywords metabolite*, metabolom* or metabonom* reveals that up to and including 2018, 2224 publications list the use of PLS as a key term, whereas the alternatives were listed < 500 times (combined number). The key to the popularity of PLS over alternative methods can be distilled into a single word—*interpretability*. Historically, the primary aim of machine learning (ML) has been accurate prediction, not statistical inference (Mendez et al. 2019a). As such, methods for statistically interpreting either the similarities between each individual metabolite profile, or the importance of individual metabolites across multiple samples, have been a secondary consideration. The ability for PLS to visualise and infer statistical confidence intervals upon the latent relationships within and between sample classes, together with the fact that a PLS model can be reduced to a simple linear regression (and thus exposed to multiple well established post-hoc statistical tests), means that it sits alone as an effective hybrid prediction-inference algorithm for high dimensional data (Eriksson et al. 2013; Wold 1975; Wold et al. 1993).

**Fig. 1:**
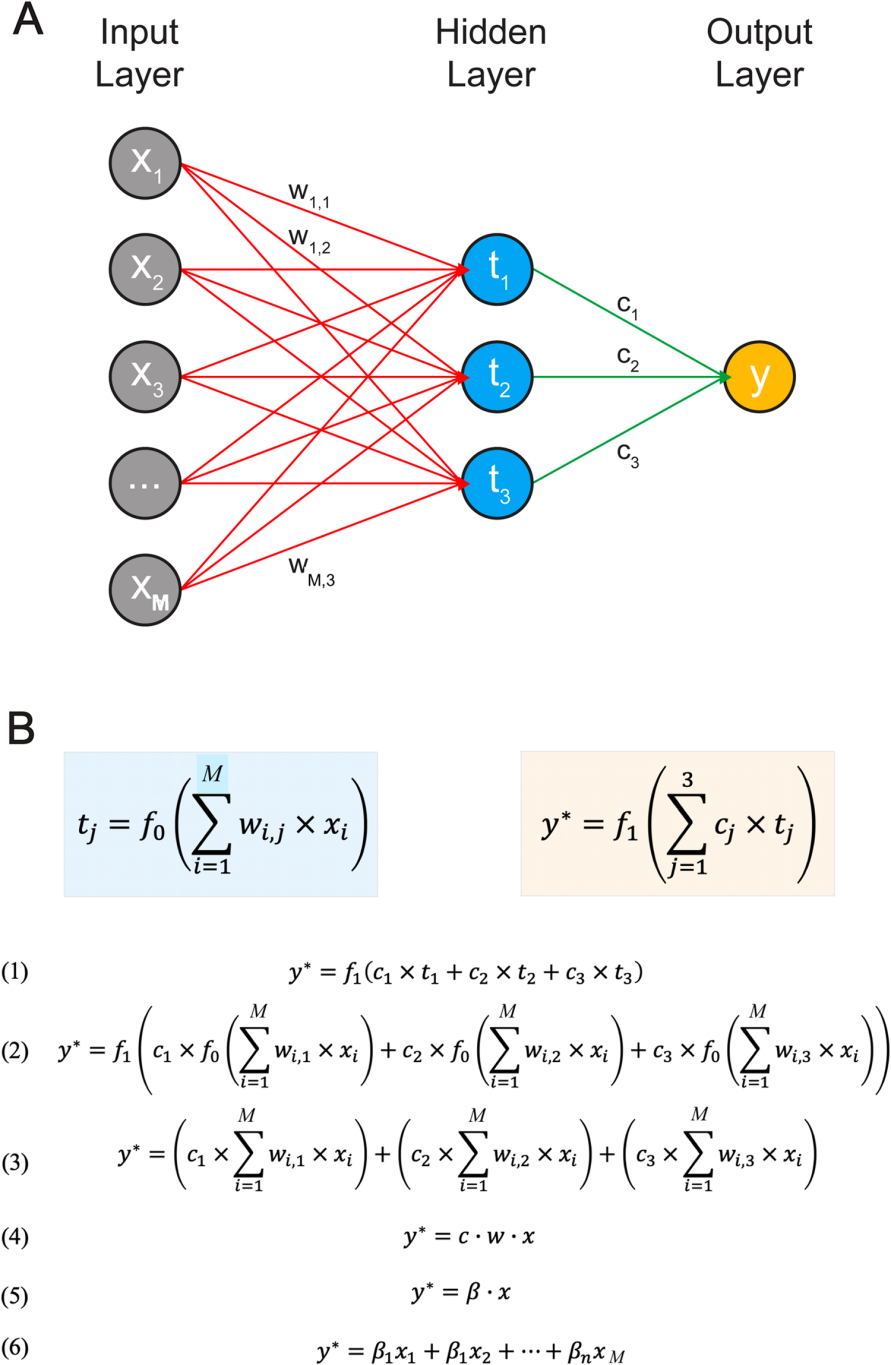
Illustration of an ANN as a regression model. **a** Network representation of a 2-layer ANN. **b** Representation of a 2-layer ANN with linear activation functions, as a set of equations, simplified to a linear regression model

Artificial neural networks (ANNs) are also of particular interest because in their simplest form, as with PLS, they can be considered as a combination of dimensionality reduction and multiple linear regression. In fact, for a linear ANN, with a single hidden layer, the only difference between ANN and PLS is the manner in which the constituent model parameters are optimised (Fig. 1). ANNs can be generally considered a projection-based method which share a *structural equivalence* with PLS (Mendez et al. 2019a). With non-linear ANNs the *projection to latent structures* ethos is preserved but now non-linear, rather than linear, latent structures can be modelled.

ANNs were first applied to metabolomic profiling ca. 1992 by Goodacre et al. (1992). At that time, due to lack of compute power and poor software availability, ANNs were very slow to train and considered difficult to interpret. As such, by the early 2000s they had been widely disregarded and relegated to an intellectual curiosity not considered able to provide meaningful biological insight (Goodacre 2003). With recent advancements in computational power, the availability of easily accessible yet powerful open-source packages (e.g. TensorFlow and PyTorch), and the general success within industry and other research fields, the reintroduction of ANNs warrants renewed investigation. We recently showed that ANNs have similar predictive ability to PLS across multiple diverse metabolomics data sets (Mendez et al. 2019c). However, within the domain of metabolomics, if ANNs are to become a truly viable alternative to PLS it will be necessary to develop similar standardised and robust methods for data visualisation, evaluation, and statistical inference (Mendez et al. 2019a).

Recently, the increased availability of well curated open-source software libraries, particularly from R and Python programming communities, has increased the availability and utility of many ML methods, including ANNs. Moreover, the massive increase in available computer power has reduced compute times such that methods previously intractable due to computational expense, such as bootstrap confidence intervals (Efron 1988), have enabled non-parametric statistical inference to be derived for previously considered uninterpretable ‘black box’ methods. This opens the door for the development of an ANN framework comparable to that of PLS-DA.

The aim of this study is to migrate the standardised *optimisation, visualisation, evaluation,* and *statistical inference* techniques commonly used in a PLS-DA binary classification over to a non-linear, single hidden layer, ANN algorithm, and then conduct a direct comparison of utility. We provide two functionally equivalent workflows (PLS-DA vs. ANN) implemented using the Python programming language, and presented as open-access Jupyter Notebooks (https://cimcb.github.io/MetabProjectionViz/). The workflows were applied to two previously published metabolomics datasets by Chan et al. (2016) and Ganna et al. (2016), but are written to be used with any data set suitably formatted following previous guidelines (Mendez et al. 2019b). Both workflows include cross-validated hyperparameter optimisation, latent variable projection scores plots, classification evaluation using receiver operator characteristic curves, bootstrap resampling for statistical inference of feature contribution and generalisability of prediction metrics.

## Methods

### Partial least squares discriminant analysis (PLS-DA)

PLS-DA (Wold 1975; Wold et al. 1993) is a widely used multivariate ML algorithm used for classifying and interpreting metabolomics data, especially applicable when the number of metabolites (independent variables) is much larger than the number of data points (samples). PLS uses the *projection to latent space* approach to model the linear covariance structure between two matrices (**X** and **Y**). If the **X** matrix is thought of as a set of *N* data points in *M*-dimensional space (where, *N* = number of samples, and *M* = number of metabolites), and **Y** is a binary vector (length *N*) describing the class of each samples (e.g. case = 1 and control = 0), and if we consider the algorithm geometrically, the PLS algorithm rotates and projects **X** into a lower *K* dimensional space (typically *K* = 2 or 3), represented by the scores matrix **T**, such that discrimination (covariance) between the two labelled groups in the subspace is maximised (Eriksson et al. 2013). For this study, PLS-DA models was optimised using the iterative SIMPLS algorithm (de Jong, 1993). **T** can be derived from **X** using Eq. (1), where **W,** the X-weight matrix, describes how the X-variables are linearly combined, or geometrically rotated, to form the score vectors, $t_{1} \,t_{2} \, \ldots \,t_{K}$.

1 $${\mathbf{T}}\,{\mathbf{ = }}\,{\mathbf{XW}}$$

The predicted classification (Y*) can then be calculated from **T** using Eq. (2), where **C** is the Y-weights matrix describing how the **Y** vector is rotated to map to the covariance described by **T**.

2 $${\mathbf{Y}}^{*\,} \, = \,{\mathbf{TC^{\prime}}}$$

These matrix equations, Eq. (1) and Eq. (2), can be combined and simplified to a single linear regression, Eq. (3), where **B**_**PLS**_ is a vector of coefficient values.

$${\mathbf{Y}}^{*\,} \, = \,{\mathbf{TC^{\prime}}}$$

$${\mathbf{Y}}^{*\,} \, = \,{\mathbf{XWC^{\prime}}}$$

3 $${\mathbf{Y}}^{*\,} \, = \,{\mathbf{XB}}_{{{\mathbf{PLS}}}}$$

This matrix equation, Eq. (3), can also be described as a single linear regression in standard form, Eq. (4), where $\beta_{0} \,...\,\beta_{N}$ is a vector of linear coefficients.

4 $$y^{*} = \beta_{0} + \beta_{1} x_{1} + \beta_{1} x_{2} + \ldots + \beta_{M} x_{M}$$

#### PLS-DA optimisation

The optimal number of latent variables, *K*, is determined such that the **T** matrix is just sufficient to accurately describe the underlying latent structure in **X** but not so large as to also model random correlation and produce a model that is a poor classification tool for new X-data (see cross-validation in Sect. 3.4). In machine learning terminology any parameter which is used to define a model’s structure, or an optimisation algorithm characteristic, is known as a *hyperparameter*. Thus, the number of latent variables is the single PLS-DA hyperparameter.

#### PLS-DA evaluation

In order to provide some level of independent model evaluation it is common practice to split the source data set into two parts: training set and test set (typically, 2/3 training and 1/3 test). Once the optimal number of latent variables has been determined using the training data only (${\mathbf{X}}_{{{\mathbf{train}}}}$ and ${\mathbf{Y}}_{{{\mathbf{train}}}}$), the resulting model**, **${\mathbf{Y}}^{*\,} \, = \,{\mathbf{XB}}_{{{\mathbf{PLS}}}}$, is then independently evaluated by applying the test data (${\mathbf{X}}_{{{\mathbf{test}}}}$; suitably transformed and scaled) to the model, ${\mathbf{Y}}_{{{\mathbf{Test}}}}^{*} \, = \,{\mathbf{X}}_{{{\mathbf{test}}}} {\mathbf{B}}_{{{\mathbf{PLS}}}}$. A measure of the predictive ability of the model can then be calculated by comparing the training prediction $({\mathbf{Y}}_{{{\mathbf{train}}}}^{*} )$ to the expected training outcome (**Y**_**train**_), and the test prediction (**Y**_test_^*^) to the expected test outcome (**Y**_**test**_).

While true effectiveness of a model can only be assessed using test data (Westerhuis et al. 2008; Xia et al. 2013), for small data sets it is dangerous to use a single random data split as the only means of model evaluation, as the random test data set may not accurately represent the training data set (Mendez et al. 2019c). An alternative is to use bootstrap resampling. Bootstrap resampling is a method for calculating confidence intervals using random sampling with replacement (DiCiccio and Efron 1996; Efron 1981, 2000). The theoretical details of this methodology are beyond the scope of this paper. Briefly, this technique allows the accurate estimation of the sampling distribution of almost any statistic using repeated random sampling. Each random sample selects ~ 2/3 of the data points (called the in-bag sample) leaving ~ 1/3 (the out-of-bag sample).

Bootstrapping can be used to calculate confidence measurements for the evaluating the optimal ML model configuration for a given metabolomics data set (Broadhurst and Kell 2006; Mendez et al. 2019b; Xia et al. 2013). A model with fixed hyperparameter values is retrained on data, randomly sampled with replacement (in-bag), and then evaluated on the unused data (out-of-bag) for *r* resamples (typically *r* = 100). The predicted outcome from each in-bag bootstrap resample as well as other outputs, including the predicted outcome, latent scores, latent loadings, and feature contribution metrics are stored after each resampling. The out-of-bag prediction of classification is also stored, as this can be considered an unbiased estimate of the model’s performance when shown new data. Using these stored outputs, 95% confidence intervals are calculated using the commonly-used bias-corrected and accelerated (BCa) method; this method adjusts the percentiles to account for the bias and skewness in the bootstrap distribution (Efron 1987). Following bootstrap resampling, a measure of generalised prediction of each model is calculated as the median and 95% confidence intervals of the in-bag and out-of-bag predictions.

#### PLS-DA visualisation

For a given PLS-DA model it is common practice to visualise the projection of **X** into the latent variable space to provide a generalised understanding of the metabolomic relationship (clustering) between individual samples before classification. For this, the scores matrix, **T**, described in Eq. (1), can be represented as a scatter plot (scores plot) such that each axis of the plot represents a column of the T-matrix. For example, a scatter plot of t_1_ vs. t_2_ will represent the projections of X onto the first two latent variables (i.e. each data point represents a projection of a given sample’s metabolite profile). It is in this latent variable space that one would expect to see different metabotypes cluster. The associated weight vectors (columns of **W**) can also be visualised individually and interpreted as an indication of how the X-variables are linearly combined to create each score vector, Eq. (5).

$$\begin{array}{*{20}c} {t_{1} = w_{0,1} + w_{1,1} x_{1} + w_{2,1} x_{2} + \ldots + w_{M,1} x_{M} } \\ {t_{2} = w_{0,2} + w_{1,2} x_{2} + w_{2,2} x_{2} + \ldots + w_{M,2} x_{M} } \\ \ldots \\ {t_{K} = w_{0,K} + w_{1,K} x_{1} + w_{2,K} x_{2} + \ldots + w_{M,K} x_{M} } \\ \end{array} \,(5)$$

For a single optimised model, latent scores plots can be generated for training, cross-validation, and test X-data sets independently. This is a useful method for determining if overtraining has occurred (see supplementary Jupyter Notebooks).

#### PLS-DA variable contribution

For PLS-DA, there are two common methods used to estimate variable contribution. First, as discussed, a PLS-DA model can be reduced to a single multiple linear regression, Eq. (3), thus feature contribution can be inferred directly from the model’s regression coefficients, **B**_**PLS**_. Second, for more of a focus on the importance of the X-variables on the latent projection, the *variable influence on projection* (VIP) scores can be calculated using Eq. (6) (Favilla et al. 2013). VIP is the weighted,$,w_{i}^{2}$ combination of the sum of squares of Y explained by each latent variable, $SSY_{i}$, normalised to the cumulative sum of square, $SSY_{cum}$,

where $M$ is the total number of metabolites, and $K$ is the total number of latent variables.

6 $${\mathbf{VIP}} = \sqrt {M \times \frac{{\mathop \sum \nolimits_{i = 1}^{K} w_{i}^{2} \times SSY_{i} }}{{SSY_{cum} }}}$$

The average VIP score is equal to 1 because the sum of squares of all VIP scores is equal to the number of variables in **X**. Thus, if all X-variables have the same contribution to the model, they will have a VIP score equal to 1. VIP scores larger than 1 indicate the most relevant variables. Bootstrap resampling (Sect. 2.1.2) can be applied to calculate 95% confidence intervals for both the **B**_**PLS**_ coefficient values and **VIP** scores, from which estimates of significant contribution to the model can be determined.

### Artificial neural network (ANN)

ANNs consist of layered weighted networks of interconnected mathematical operators (neurons). The most prevalent ANN is the feed-forward neural network. Here, each neuron acts as a weighted sum of the outputs of the previous layer (or input data) transformed by an activation function (typically linear or logistic function). This is described in Eq. (7), using notation from Fig. 1a, where ${t}_{j}$ is the output for the *j*^*th*^ neuron in the hidden layer, ${f}_{0}$ is the activation function, $x$ is a vector of input variables (x_1_, x_2_, …, x_M_), ${w}_{i,j}$ is the weight from input variable, x_i_, to the neuron, and *w*_*0,j*_ is a constant offset value.

7 $$t_{j} = f_{0} \left( {w_{0,j} + \mathop \sum \limits_{i = 1}^{M} w_{i,j} \times x_{i} } \right)$$

A neuron with a linear activation function connected to multiple input variables is mathematically equivalent to a linear regression with multiple independent variables, Eq. (8), where w_0,j_ … w_N,j_ is a vector of linear coefficients.

8 $$t_{j} = w_{0,j} + w_{1,j} x_{1} + w_{2,j} x_{2} + \cdots + w_{M,j} x_{M}$$

A neuron with a logistic activation function, f_0_ (), is equivalent to the multivariate logistic regression describe in Eq. (9).

9 $$t_{j} = \frac{1}{{1 + e^{{ - \left( { w_{0,j} + \mathop \sum \nolimits_{i = 1}^{M} w_{i,j} \times x_{i} } \right)}} }}$$

An ANN with a single linear hidden layer and a single linear output neuron is mathematically equivalent to a PLS-DA model (Fig. 1). Replacing all the linear neurons with logistic neurons in the two-layer ANN results in a complex non-linear projection-based discriminant model. For this study, we use a two-layer ANN with logistic activation functions in both layers.

#### ANN optimisation

During ANN training, the interconnection weights between each layer of neurons are optimised using an iterative algorithm known as *back-propagation*. This algorithm has been described in detail elsewhere (Bishop 1995). The effectiveness of this optimisation method is dependent on a set of *hyperparameters.* A two-layer feedforward ANN has 5 hyperparameters: 1 parameter to determine the model structure, the *number of neurons* in the hidden layer (equivalent to number of latent variables) and 4 parameters that characterise the learning process. These determine the rate and momentum of traversing local error gradients (specifically *learning rate*, *momentum*, and *decay* of the learning rate over time) and the number of times the back-propagation is applied to the ANN (the number of training *epochs*). For this study, preliminary explorative analysis indicated that hyperparameters: *momentum*, *decay*, *epochs* could be set to a constant value (*0.5*, *0* and *400* respectively) with little variation on performance. This reduced the number of tuneable hyperparameters to: (i) the *number of neurons in the hidden layer*, and (ii) the *learning rate*.

#### ANN evaluation

Model evaluation using a test set and model evaluation using bootstrap resampling is identical to that described in Sect. 2.1.2. except replacing the PLS-DA prediction, Y^*^, with the ANN equivalent.

#### ANN visualisation

For an equivalent representation of the PLS-DA projection to latent space, we provide a projection to neuron space. Each hidden neuron represents a transformed weighted sum of the X-variables (Eq. 7). Thus, for each pairwise combination of neurons, plotting the weighted sum before transformation provides a similar means to PLS-DA for visualising and interpreting any clustering between individual samples before classification. Similarly, associated weight vectors can also be visualised individually and interpreted as an indication of how the X-variables are linearly combined to create each neuron scores vector before transformation.

#### ANN variable contribution

For ANN, several variable contribution metrics have been proposed (Olden et al. 2004); however, the two most comparable metrics to the PLS-DA **B**_**PLS**_ coefficients and VIP scores are the Connection Weight Approach (CWA) (Olden and Jackson 2002) and Garson’s Algorithm (GA) (Garson 1991), respectively. Similar to **B**_**PLS**_, for a two-layer ANN with linear activation functions (Fig. 1b), feature contribution can be inferred directly from a model’s linear coefficients, **B**_**ANN**_, as shown in Eq. (10), where **C** is the weights for the hidden-output layer, and **W** is the weights for the input-hidden layer.

10 $${\mathbf{CWA}} = {\mathbf{B}}_{{{\mathbf{ANN}}}} = {\mathbf{CW}}$$

This equation can be used to calculate variable contribution for two-layer non-linear ANNs, renamed as CWA, and describes *relative* (and *directional*) metabolite contribution.

While VIP may not be directly applied to non-linear ANNs, a similar measure of weighted *absolute relative* contribution of each metabolite per neuron can be calculated using Garson’s Algorithm (Garson 1991). First, absolute *CWA*_*i,j*_values are calculated across the network by multiplying each neuron input weight, *w*_*i,j*_, to the corresponding output weight,*c*_*j*_and converting to an absolute value.

11 $$\left| {CWA_{i,j} } \right| = \left| {w_{i,j} \times c_{j} } \right|$$

Second, as shown in Eq. (12), for each hidden neuron the total absolute connection weight value is calculated, where $M$ is the total number of metabolites.

12 $$\left| {CWA_{j} } \right| = \mathop \sum \limits_{i = 1}^{M} \left| {CWA_{i,j} } \right| { }$$

Then, the overall contribution for each input variable, *GA*_*i*_, is calculated as shown in Eq. (13), where $K$ is the total number of hidden layer neurons.

13 $$GA_{i} = \mathop \sum \limits_{j = 1}^{K} \left( {\frac{{\left| {CWA_{i,j} } \right|}}{{\left| {CWA_{j} } \right|}}} \right)$$

Unlike VIP there is no general threshold of importance for Garson’s Algorithm, so we propose using the average GA score as a comparable equivalent to indicate metabolites of importance in the model.

### Computational workflow

**Fig. 2:**
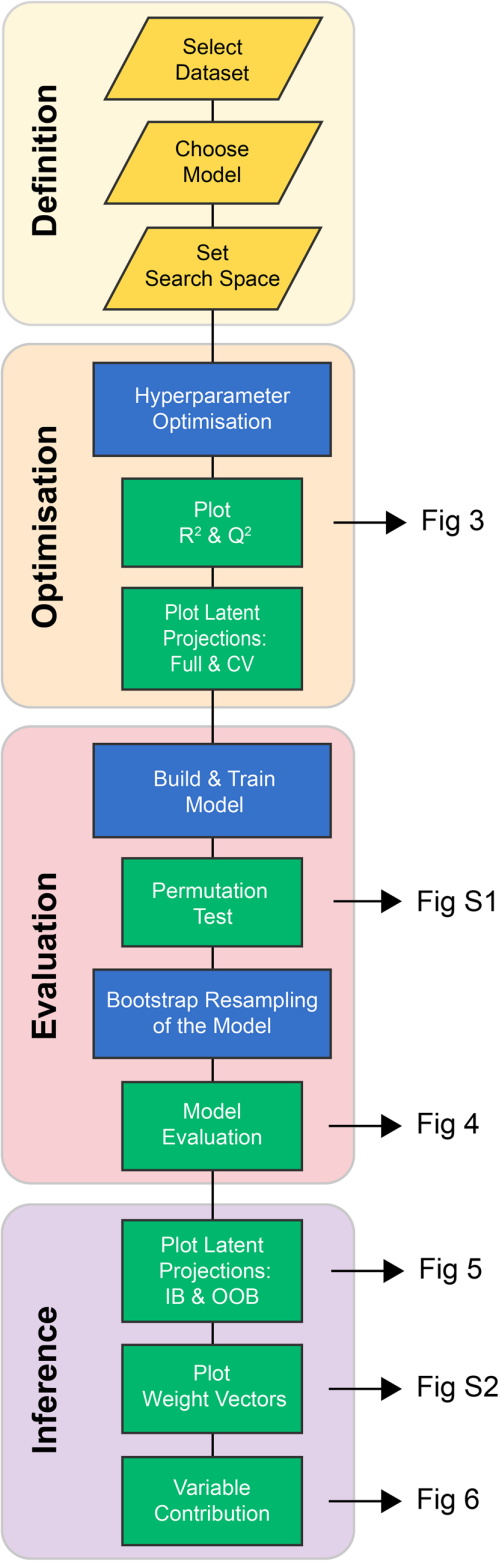
Data analysis workflow. Flowchart of the data analysis workflow used for the PLS and ANN methods. Arrows identify the figure corresponding to the respective workflow step

The standard workflow for the PLS visualisation and interpretation, and the proposed equivalent ANN visualisation and interpretation is described in Fig. 2. Both the PLS-DA and ANN workflows were implemented in the Python programming language using a package called ‘cimcb’ (https://github.com/CIMCB/cimcb) developed by the authors. This package contains tools for the analysis and visualisation of untargeted and targeted metabolomics data. The package is based on existing well curated open-source packages (including *numpy* (Kristensen and Vinter, 2010), *scipy* (Virtanen et al. 2019), *bokeh* (Bokeh Development Team 2018), *keras* (Chollet 2015), *pandas* (McKinney 2010), s*cikit-learn* (Pedregosa et al. 2011*)*, and *Theano* (Theano Development Team^2016^)). It utilises these packages through *helper functions* specifically designed to simplify the application to metabolomics data, following guidelines previously described (Mendez et al. 2019b).

Each step of the respective PLS-DA and ANN workflow is described in detail in the associated Jupyter Notebook file (included in supplementary material and https://cimcb.github.io/MetabProjectionViz/). The method of embedding explanatory text within functional code and visualisations follows previously published guidelines (Mendez et al. 2019b). The generic workflow is now briefly described.

#### Prepare data

For an adequate comparison of visualisation and interpretation methods, across PLS and ANN, it was important that identical data were used in both models. The **X** matrix of metabolite concentrations, and associated **Y** vector of classification labels (case = 1, control = 0) were extracted from the excel spreadsheet. Metabolites in **X** were included for modelling if they had a QC relative standard deviation (RSD_QC_) < 20% and < 10% missing data (Broadhurst et al. 2018). The datasets were split using a ratio of 2:1 (2/3 training, 1/3 test) using stratified random selection. After splitting the data into training and test sets, the columns of **X** were natural log transformed, mean centred, and scaled to unit variance with missing values imputed using k-nearest neighbour prior to modelling following standard protocols for metabolomics (Broadhurst and Kell 2006). The means and standard deviations calculated from the training set were applied to scale the test set data.

#### Hyperparameter optimisation

For both PLS-DA and ANN algorithms the optimal hyperparameter values were determined using 5-fold cross-validation (CV) with 10 Monte Carlo repartitions (Broadhurst and Kell 2006; Hastie et al. 2009; Xia et al. 2013). For the PLS-DA workflow, a linear search was used to optimise the number of latent variables (1 to 6). For the ANN workflow, a grid search was used to optimise the number of neurons (2 to 6) and the learning rate (0.001 to 1). The optimal hyperparameter values were determined by evaluating plots of *R*^2^ and *Q*^2^ statistics. Two plots were generated: (i) a standard *R*^2^and *Q*^2^ plot against hyperparameter values, and (ii) an alternative plot of $\left| {R^{2} - Q^{2} } \right| vs. Q^{2}$. Using the later plot, the optimal hyperparameter was selected at the point of inflection of the outer convex hull. The area under the receiver operating characteristic curve (AUC) is a recommended alternative non-parametric measure of classification performance (Szymańska et al. 2012), thus equivalent plots of *AUC*_*Full*_ and *AUC*_*cv*_ metrics are also generated for comparison.

#### Permutation test

Following hyperparameter optimisation, a permutation test was applied to the optimal model configuration. In a permutation test, the expected outcome label is randomised (permuted), and the model with fixed hyperparameter values is subsequently trained and evaluated (Lindgren et al. 1996). For both PLS-DA and ANN, this process was repeated (n = 100) using fivefold CV to construct a distribution of the permuted model statistics. While *R*^2^and *Q*^2^ statistics are commonly used in permutation testing (Eriksson et al. 2013), *AUC*_*Full*_ and *AUC*_*cv*_ metrics were also included for ANNs, given its common usage as a measure of non-linear classification performance.

#### Model evaluation using test set

As previously described in Sect. 2.1.2, the measure of the predictive ability of the model using a test set is calculated by comparing the training score (${\mathbf{Y}}_{{{\mathbf{train}}}}^{*}$) to the expected outcome (**Y**_**train**_) classification, and the test score (${\mathbf{Y}}_{{{\mathbf{test}}}}^{*}$) to the expected outcome (**Y**_**test**_) classification. This is visualised using three plots:

1. A violin plot that shows the distribution of the predicted score, by outcome, for the training and test set.
2. A probability density plot that shows the distribution of the predicted score, by outcome, for the training and test set via overlapping probability density functions.
3. A receiver operator characteristic (ROC) curve of the training and test sets.

#### Model evaluation using bootstrap resampling

Model evaluation using bootstrap resampling is described in Sect. 2.1.2. Following bootstrap resampling (n = 100), a measure of generalised prediction of each model is calculated and visualised using the protocol described in 2.3.4, except this time presenting the 95% confidence intervals of the 100 in-bag and out-of-bag predictions.

#### Model visualisation: scores plot & weights plot

Pairwise latent variable scores plots and associated weight vector plots are also provided. The scores plots are similar in construction to those generated during hyperparameter optimisation, except they are based on the in-bag and out-of-bag scores averaged across repeated prediction for each sample (aggregate score). 95% confidence intervals for each class are calculated using standard parametric methods. The 95% confidence intervals for each weight vector plots were constructed using the distribution of each weight variable across the 100 bootstrap resampled models. Any metabolite weight with a confidence interval crossing the zero line (coloured blue) are considered non-significant to the latent variable (or neuron).

#### Variable contribution plots

The **B**_**PLS**_ coefficients and VIP scores for the PLS models were calculated using the methods described in Sect. 2.1.4. The CWA and Garson scores were calculated for the ANNs using the methods described in Sect. 2.2.4. There metrics were also applied to all 100 models of each type generated during the bootstrap resampling. Variable contribution plots were constructed. The 95% confidence intervals for each vector plots were calculated using the distribution of each variable’s metric across the 100 bootstrap resampled models. Any metabolite weight with a confidence interval crossing the zero line are considered non-significant to the latent variable (or neuron).

The variable contribution metrics for each model type was compared and contrasted through visual inspection of a scatter plots of **B**_**PLS**_*vs***. CWA**_**ANN**_ and of **VIP**_**PLS**_* vs.***Garson**_**ANN**_ scores, and by calculating the associated Pearson’s correlation coefficient.

## Results

### Datasets

In this study, a previously published dataset by Chan et al. (2016) was used to illustrate the standardised PLS workflow and the proposed equivalent ANN workflow. This urine nuclear magnetic resonance (NMR) dataset, comprised of 149 metabolites, is publicly available on *Metabolomics Workbench* (Study ID: ST0001047). For the work described herein a binary classification was performed: gastric cancer (n = 43) vs. healthy controls (n = 40).

The computational libraries developed for this study require data to be converted to a standardised format using the *tidy data* framework (Wickham, 2014). This standardised format has been previously described (Mendez et al. 2019b, 2019c), and allows for the efficient reuse of these workflows for other studies. To demonstrate this, we include the application of the identical workflows and visualisation techniques to a second previously published dataset (Ganna et al. 2016) as a supplementary document. This plasma liquid chromatography-mass spectrometry (LC–MS) dataset, comprised of 189 named metabolites, is publicly available on *MetaboLights* (Study ID: MTBLS90), and for this study, samples were split into two classes by sex: males (n = 485) and females (n = 483). This dataset did not report QC measurements and therefore the data cleaning step was unable to be performed.

**Fig. 3:**
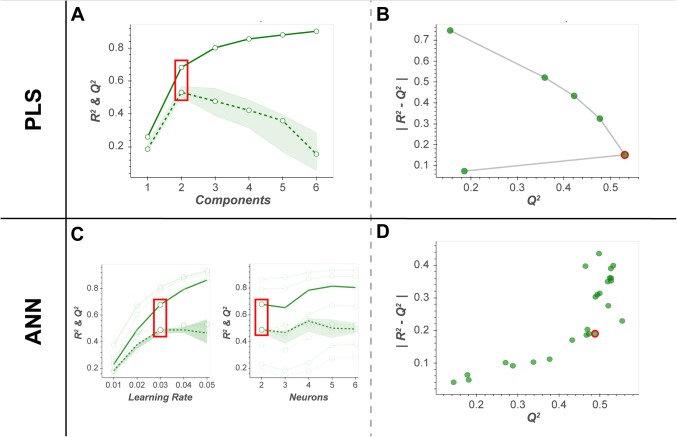
Hyperparameter optimisation. Plots of *R*^2^ and *Q*^2^ statistics; red circle, optimal hyperparameter value(s). **a** & **c** Standard *R*^2^ and *Q*^2^ vs hyperparameter values plot for PLS and ANN, respectively. Solid line, *R*^2^; dashed line, *Q*^2^. **b** & **d** The alternate $\left| {R^{2} - Q^{2} } \right| vs. Q^{2}$ plot for PLS and ANN, respectively. The optimal hyperparameters shown in panel **c** were identified using the plot in panel **d**

**Fig. 4:**
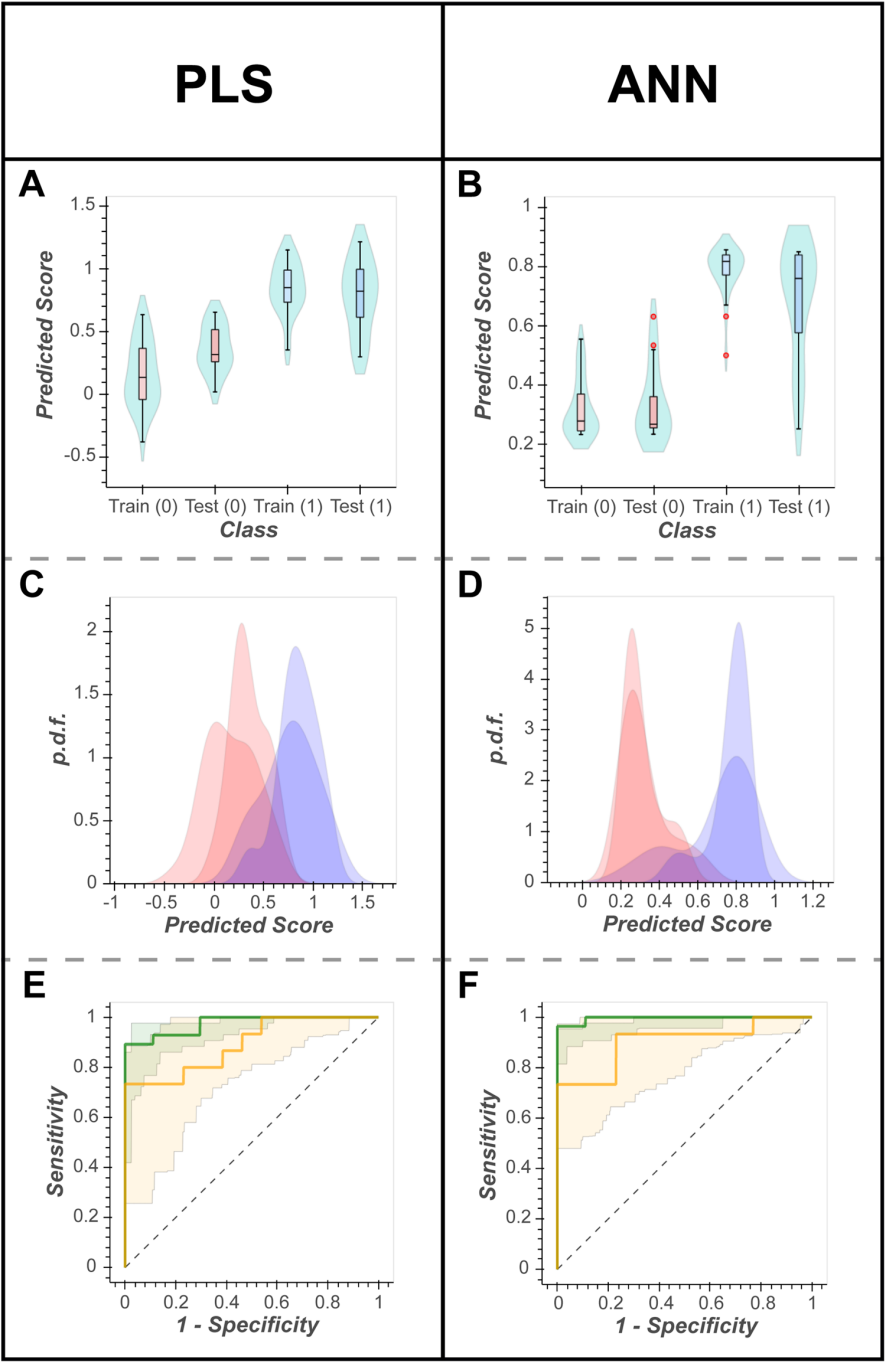
Visualisations of model evaluation. Predicted scores (train and test) split into the respective binary classification, visualised in three different ways. **a**, **b** Violin plots; **c**, **d** probability distribution function (pdf) plots. Red, healthy controls (control); blue, gastric cancer (case). **e**, **f** ROC curves with 95% CIs derived from 100 iterations of bootstrap resampling. Green line predicted scores for training set; green 95% CIs, IB predictions; yellow line, prediction scores for test set; yellow 95% CIs, OOB predictions. PLS-DA AUC_Train_ = 0.97, AUC_Test_ = 0.89, AUC_IB_ = 0.92–0.99, AUC_OOB_ = 0.72–0.98. ANN AUC_Train_ = 1.00, AUC_Test_ = 0.90, AUC_IB_ = 0.95–0.99, AUC_OOB_ = 0.77–1.00

**Fig. 5:**
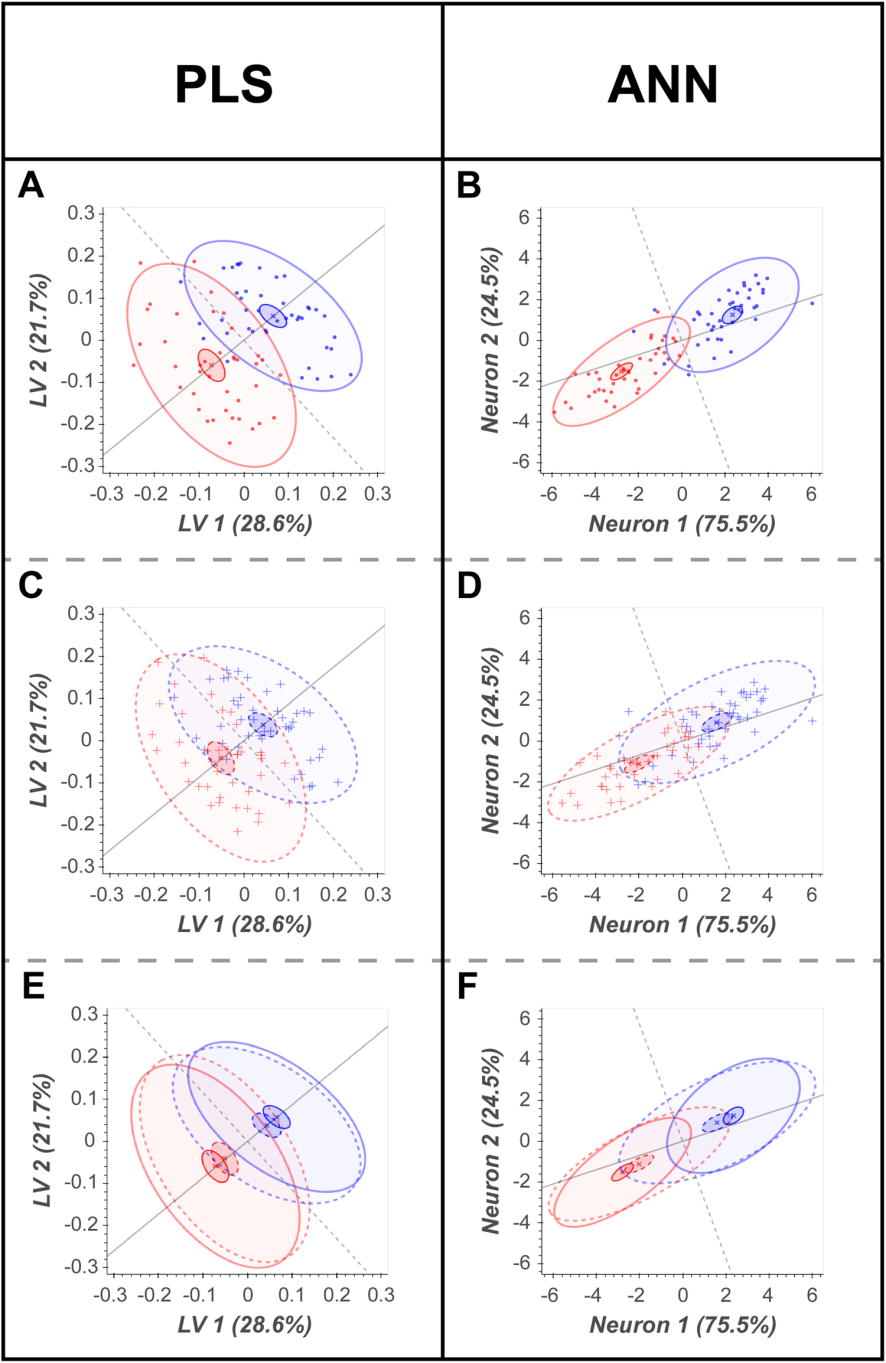
Bootstrap projection (scores) plots. Projection plots show LV2 vs LV1 for PLS and Neuron 2 vs Neuron 1 for ANN. **a**, **b** projected scores of the median IB; **c**, **d** projected scores for median OOB; **e**, **f** median IB and median OOB scores overlaid. Red, healthy control (control); blue, gastric cancer (case). Inner ellipses, 95% CI of the mean; outer ellipses, 95% CI of the population. Solid lines, IB predictions; dashed lines, OOB predictions

**Fig. 6:**
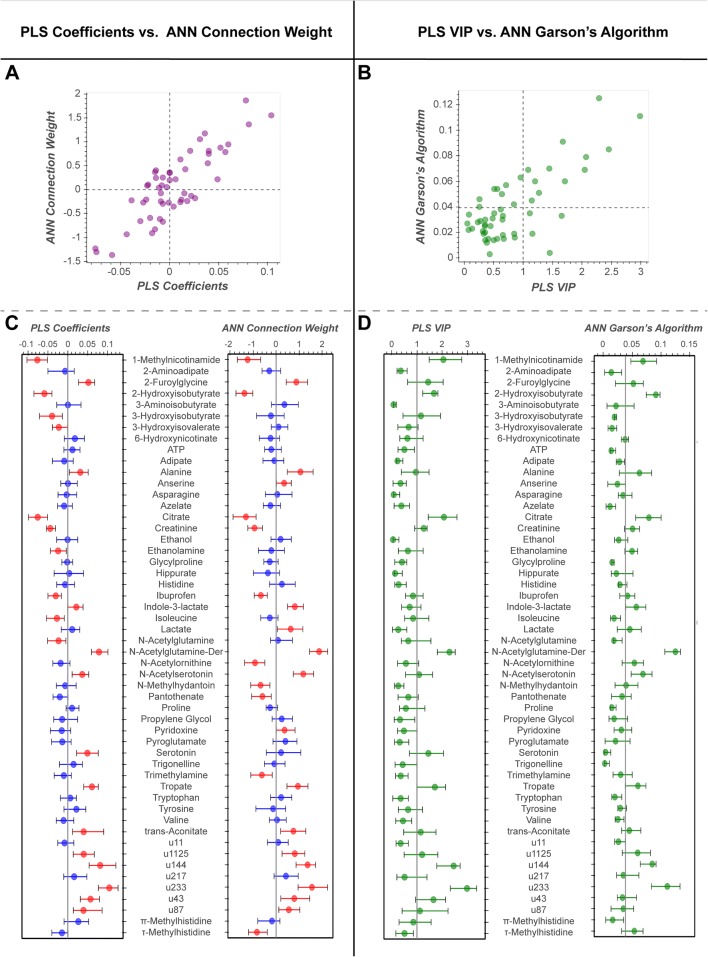
Variable contribution. Visualisation of variable contribution for PLS (coefficients and VIP) and ANN (CWA and Garson’s algorithm). **a** Scatterplot of *ANN*_*CWA*_ vs. ***B***_*PLS*_, Pearson’s r = 0.85 (p-value = 2.79e^−15^). **b** Scatterplot of *Garson*_*ANN*_ vs. *VIP*_*PLS*_, Pearson’s r = 0.75 (p-value = 1.33e^−10^). Dashed lines at respective “importance” cut-off: *Garson*_*ANN*_ = 0.038, *VIP*_*PLS*_ = 1.00. **c** Median (and 95% CI) ***B***_*PLS*_ (left) and *ANN*_*CWA*_ (right). Blue, contribution not significant based on 95% CIs; red, contribution significant based on 95% CIs. **d** Median (and 95% CI) *VIP*_*PLS*_ (left) and *Garson*_*ANN*_ (right)

Following data cleaning, for the urine NMR gastric cancer data set 52 metabolites were included in data modelling (case = 43 vs. control = 40). Figures 3, 4, 5 and 6 (and Supplementary Figs. S1-2) show the optimisation, visualisation, evaluation and statistical inference for the PLS-DA compared to the ANN algorithms. Similar plots are provided in supplementary documentation for the plasma LC–MS data set (males = 485 vs. females = 483). All 4 workflows are also available as interactive Jupyter notebooks (https://cimcb.github.io/MetabProjectionViz/), either to be downloaded or to be run in the cloud through mybinder.org. See Mendez et al. (2019b) for guidance.

### Model optimisation

Using the = $\left| {R^{2} - Q^{2} } \right| vs. Q^{2}$ plot, both the number of latent variables (LV = 2; Fig. 3b) and ANN hyperparameters (learning rate = 0.03 & hidden neurons = 2; Fig. 3d) were clearly interpretable. These findings were verified using permutation testing (Supplementary Fig. 1).

### Model evaluation and visualisation

Strategies for model evaluation and visualisation were successfully transferred from PLS-DA to ANNs. For both example data sets the ANN model performed slightly better than the PLS-DA for both the training and test data sets (Fig. 4). Both models somewhat overtrained despite rigorous cross-validation. For the PLS-DA model the AUC_Train_ = 0.97 and the AUC_Test_ = 0.89. For the ANN model the AUC_Train_ = 1.00 and AUC_Test_ = 0.90. Bootstrap remodelling also showed similar results. The PLS-DA model had an in-bag area under the ROC curve (AUC) with 95% CI of 0.92–0.99. Similarly, the ANN produced an in-bag AUC with 95% CI of 0.95–0.99. The out-of-bag predictions showed that both models overtrained with out-of-bag AUC 95% CI of 0.72–0.98 (PLS-DA) and 0.77–1.00 (ANN). The bootstrap projections confirmed these findings and illustrated that the models were still able to project significant mean differences between classes, for both the in-bag and out-bag projections (Fig. 5).

### Model inference

Feature contribution was determined by calculating bootstrap confidence intervals for the model coefficients ***B***_***PLS***_ (or equivalent *CWA*_*ANN*_) and of the *VIP*_*PLS*_ (or equivalent *Garson*_*ANN*_). Across the two models, ***B***_***PLS***_ and *CWA*_*ANN*_ showed a high degree of correlation (Fig. 6a; Pearson’s r = 0.85, p = 2.8 × 10^−15^). Twenty-three metabolites significantly contributed to the PLS-DA model and 25 metabolites significantly contributed to the ANN model, with an overlap of 17 metabolites being significant in both models (Fig. 6a). The *VIP*_*PLS*_ and *Garson*_*ANN*_ values showed a reduced, but still significant, degree of correlation with each other (Fig. 6b; Pearson’s r = 0.75, p = 1.33 × 10^−10^). Based on median values alone (Fig. 6b), 12 metabolites were deemed as “important” across both models and an additional 12 metabolites were “important” in one, but not both models. When taking into consideration bootstrapped confidence intervals (Fig. 6d) *VIP*_*PLS*_ and *Garson*_*ANN*_ yielded 7 and 8 “important” metabolites, respectively. Six metabolites deemed “important” by *Garson*_*ANN*_ were also deemed important by *VIP*_*PLS*_. Although mathematical calculations for variable contribution were different for the two models, Fig. 6 shows that the overall visualisation strategy was transferrable.

## Discussion

The migration of the PLS-DA optimisation, evaluation, and interpretation workflow to a single hidden layer ANN was successful. The strategy for visualising hyperparameter optimisation was adapted to the $\left| {R^{2} - Q^{2} } \right| vs. Q^{2}$ plot (Fig. 3c–d) and readily employable to both model types. Not only did it allow for simultaneous interpretation of 2 hyperparameters (ANNs), but it provides an alternate interpretation strategy for PLS-DA optimisation if the standard *R*^2^ and *Q*^2^ vs hyperparameter value plot is ambiguous. Model evaluation and projection (scores) plots were directly transferrable from PLS-DA to ANNs. Projecting the neuron weights (in place of latent variables) before the transfer function allows for a comparative and clear visual disruption of sample similarity. The bootstrap resampling/remodelling enabled both the PLS-DA and ANN models’ predictions to be interpreted with statistical rigor. Both models had similar performance, but as described (and expected) in the bootstrap projections (Fig. 5) and loadings (Supplementary Fig. S2).

*CWA* and *Garson* provided suitable variable contribution metrics for the ANN model. The surprising similarity between ***B***_***PLS***_ and *CWA*_*ANN*_, and *VIP*_*PLS*_ and *Garson*_*ANN*_ indicates the validity of both *CWA*_*ANN*_ and *Garson*_*ANN*_ as methods of determining feature importance. These findings are validated by the second study (supplementary documentation). It is important to note that no one ML method will be superior for identifying the most biological plausible metabolites. The high level of overlap between comparable variable contribution methods, in these results, suggest that deviations are likely random false discoveries due to lack of power (as reflected in the 95% CIs are how close they are to the zero line). As the cut-off for both *VIP* and *Garson*_*ANN*_ are not statistically justified limits (Tran et al. 2014), we recommend opting for ***B***_***PLS***_ for PLS and *CWA*_*ANN*_for ANN, and using the 95% CI from bootstrap resampling to determine statistically significant metabolites.

As a side note, it is worth discussing two additional points. First, there is an advantage of using bootstrap resampled predictions and projections once the optimal hyperparameters are fixed. This is particularly important if the sample size is small and there may be large differences in results depending on how the samples are split into training and test sets. The out-of-bag predictions provide an unbiased estimate of model performance, and the averaged out-of-bag projections a more realistic estimate of generalised class-based cluster similarity. Bootstrapping can also aid in preventing false discoveries regarding metabolite significance, as the resulting 95% CIs will identify metabolites with unstable contributions to the model. Second, model outcomes and resulting interpretations can affected by the quality of the input data. We have previously shown that PLS and ANNs show similar predictive ability, when using the same input data, and that sample size is an important determinant of model stability (Mendez et al. 2019c). However, to our knowledge, an extensive comparison of different data cleaning (Broadhurst et al. 2018), pre-treatment (van den Berg et al. 2006), and imputation (Di Guida et al. 2016; Do et al. 2018) procedure options has not been performed for ANNs. As such, individual users should consider and test these effects prior to modelling their own data.

## Conclusion and future perspectives

We have shown that for binary discrimination using metabolomics data it is possible to migrate the workflow from PLS-DA to a single hidden layer non-linear ANN. For the two presented examples the ANN does not perform any better than PLS-DA, and based on coefficient plots there is very similar feature contribution. However, these results show that ANNs can be evaluated alongside PLS-DA for any data set (using the provided Jupyter notebooks it is possible to evaluate any binary classification data set provided it is formatted appropriately before uploading). If a highly non-linear relation should arise, then ANN may be a better approach to PLS. This remains to be proven.

More importantly these results open the door to investigating more complex models. As discussed previously (Mendez et al. 2019a), an area of increasing interest to the metabolomics community is multi-block data integration (e.g. multi-omic or multi-instrument). Currently, methods employed are based on hierarchical application of multiple linear projection models. For example, OnPLS (Löfstedt and Trygg, 2011; Reinke et al. 2018) is a combinatorial amalgamation of multiple PLS models, and Mixomics (Rohart et al. 2017) is a stepwise integration of canonical correlation analysis and sparse PLS. The inherent flexibility of ANN architecture allows complex relationships to be combined into a single model. It may be possible to build an ANN to combine multiple data blocks into a single model without resorting to over-simplified data concatenation. For these types of models to be useful will be necessary to incorporate feature importance, and interpretable visualisation strategies. The work presented here is a first step to applying statistical rigor and interpretability to more complex ANN models.

## Electronic supplementary material

Below is the link to the electronic supplementary material.

Supplementary file1 (DOCX 11914 kb)

## Data Availability

The metabolomics and metadata used in this paper were retrieved Metabolomics Workbench (https://www.metabolomicsworkbench.org/) Study ID: ST0001047, and from Metabolights (https://www.ebi.ac.uk/metabolights/) study identifier: MTBLS90. This data were converted from the original data format to a clean format compliant with the Tidy Data framework, this is available at the CIMCB GitHub project page: https://github.com/CIMCB/MetabProjectionViz.

## Software availability

All software developed for this paper is available at the CIMCB GitHub project page: https://github.com/CIMCB.
