# Supplementary material for: Migrating from partial least squares discriminant analysis to artificial neural networks: a comparison of functionally equivalent visualisation and feature contribution tools using jupyter notebooks
Source: Metabolomics. 2020 Jan 21;16(2):17. doi: 10.1007/s11306-020-1640-0 (PMC6974504; doi:10.1007/s11306-020-1640-0)
Supplement: Supplementary file 1 — Supplementary file1 (DOCX 11914 kb) [file 11306_2020_1640_MOESM1_ESM.docx]

Migrating from Partial Least Squares Discriminant Analysis to Artificial Neural Networks: A Comparison of Functionally Equivalent Feature Importance and Visualisation Tools using Jupyter Notebooks.

**Authors**

Kevin M Mendez^1^, David I Broadhurst^1*^, Stacey N Reinke^1*^

^1^Centre for Integrative Metabolomics & Computational Biology, School of Science, Edith Cowan University, Joondalup, 6027 Australia

*Corresponding authors:

email: d.broadhurst@ecu.edu.au, stacey.n.reinke@ecu.edu.au

phone: +61 (0)8-6304-2705

**ORCIDs:**

Kevin M Mendez: 0000-0002-8832-2607

David I Broadhurst: 0000-0003-0775-9581

Stacey N Reinke: 0000-0002-0758-0330

List of supplementary html files:

1. PLSDA_ST001047.html
2. ANNSigSig_ST001047.html
3. PLSDA_MTBLS90.html
4. ANNSigSig_MTBLS90.html

Supplementary figures: pages 2-3


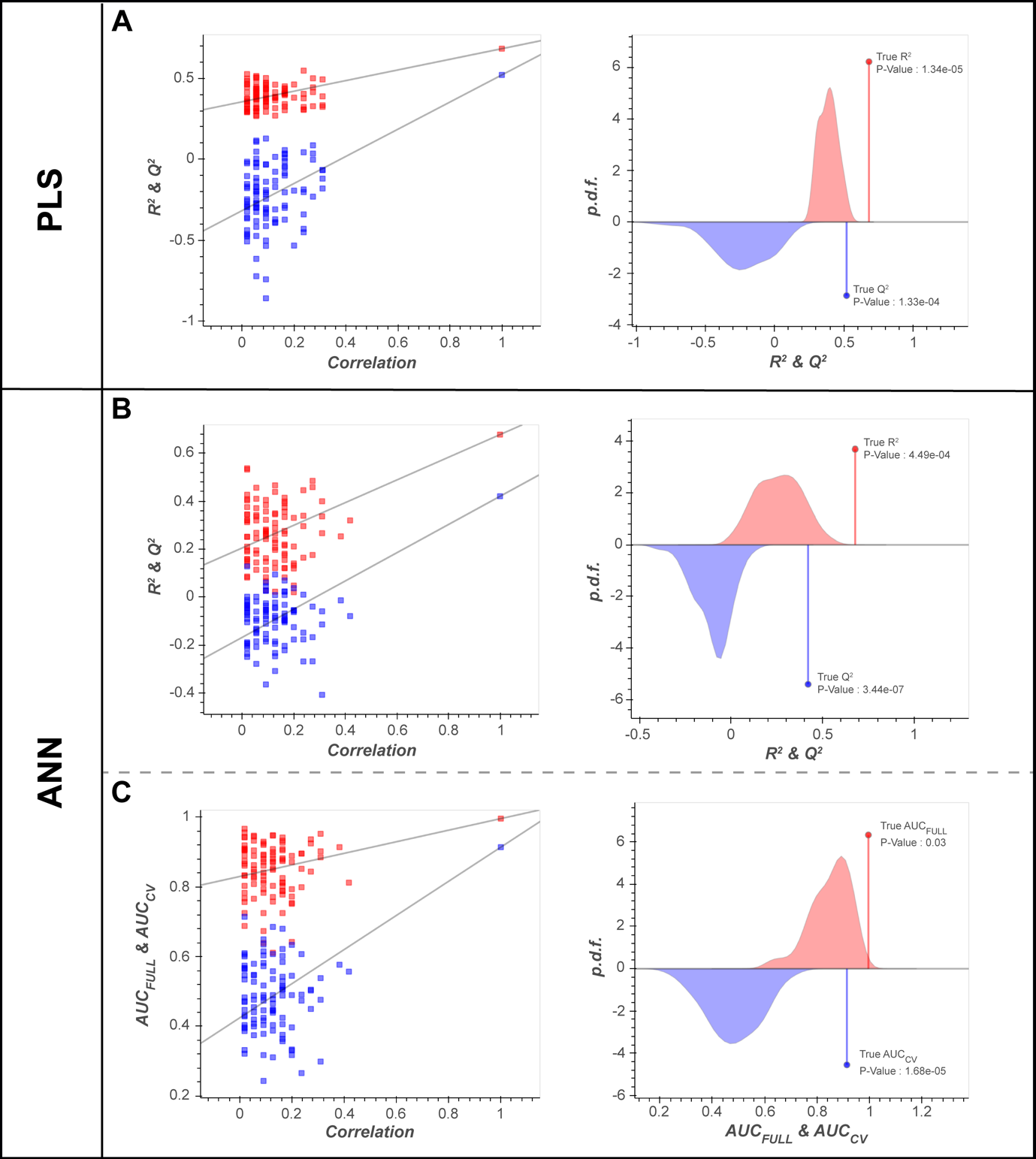


**Supplementary Figure 1: Permutation Test.** R^2^ and Q^2^ against correlation between permuted outcomes and original outcomes (left), and probability density functions for R^2^ and Q^2^, with the R^2^ and Q^2^ values of the model trained on the original data presented as a ball-and-stick, and p-values from a one-tailed t-test (right). **a** Permutation test figures for PLS. **b** Permutation test figures for ANN. **c** Permutation test figure for ANN using AUC_FULL_ and AUC_CV_ as an alternative metric to R^2^ and Q^2^.


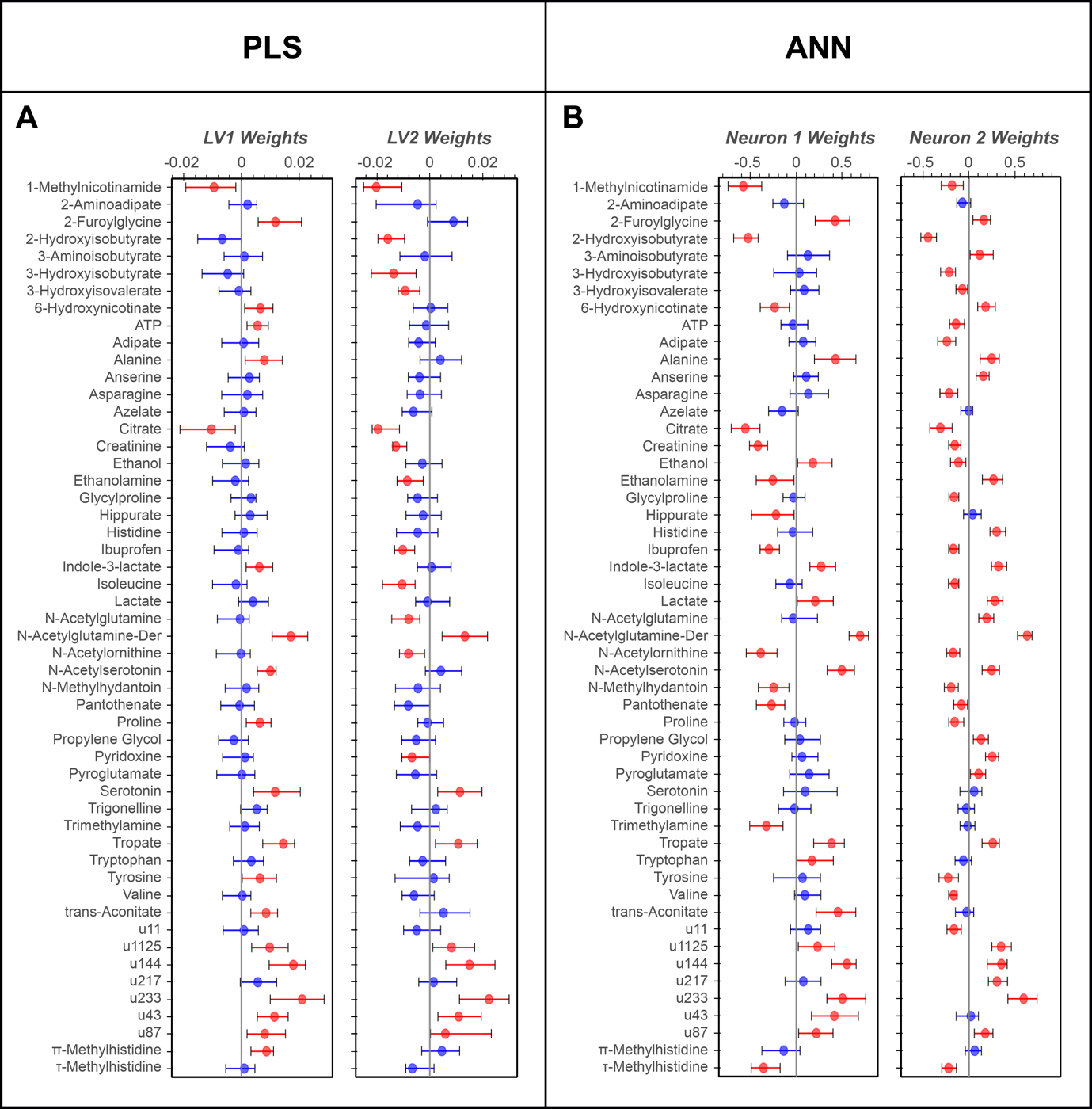


**Supplementary Figure 2: Bootstrap weight vectors.** Median (and 95% CI) weight vectors of metabolites calculated using BCa (n=100). Red, significant contribution; blue, no significant contribution. **a** LV1 and LV2 weight vectors for PLS. **b** Neuron 1 and neuron 2 weight vectors for ANN.
